# A nomogram based on hematological markers to predict radiosensitivity in patients with esophageal squamous cell carcinoma

**DOI:** 10.1097/MD.0000000000033282

**Published:** 2023-03-17

**Authors:** Lijun Sun, Peng Wei, Shuang Ge, Jie Zheng, Shucheng Ye, Yanhui Zhang

**Affiliations:** a Affiliated Hospital of Jining Medical University, Jining, China.

**Keywords:** esophageal squamous cell carcinoma, inflammation markers, radiotherapy, tumor responses

## Abstract

This study aimed to determine the predictive value of pretreatment levels of hematological markers on the radiosensitivity of patients with esophageal squamous cell carcinoma (ESCC). The specific hematological markers assessed included total lymphocyte count (TLC), neutrophil count, platelet count, monocyte count, neutrophil-lymphocyte ratio (NLR), platelet-lymphocyte ratio (PLR), and lymphocyte-monocyte ratio (LMR). A total of 353 ESCC patients who received radiotherapy (RT) alone or concurrent RT between 2015 and 2019 were reviewed. Pretreatment levels of hematological markers (NLR, PLR, LMR, and TLC) were used to assess the radiosensitivity of individual patients. Receiver operating characteristics curves were used to determine optimal cutoff values. Multivariate logistic models with radiosensitivity were established with meaningful results used for univariate analyses. Finally, a nomogram was developed and validated from the calibration curve and concordance index. One month after RT, 121 (34.3%) cases were shown to have low levels of radiosensitivity based on hematological markers. Univariate analyses showed that NLR, PLR, LMR, and TLC were associated with high levels of radiosensitivity (all markers, *P* < .05). Due to the collinearity between NLR, PLR, and LMR, these markers were separately evaluated by multivariate analysis. Multivariate analysis showed that high pretreatment NLP and PLR were independently associated with high radiosensitivity. In contrast, high pretreatment LMR and TLC were independent biomarkers associated with lower radiosensitivity. The concordance index of the nomogram was 0.737, and the calibration curves predicted by the nomogram were highly consistent with the observed experimental findings. Pretreatment hematologic markers (NLR, PLR, LMR, and TLC) can be used to predict the radiosensitivity of patients with ESCC accurately.

## 1. Introduction

Esophageal cancer (EC) is one of the most common malignancies in the world. In 2018, EC was found to be the seventh most prevalent malignancy and the sixth most common cause of cancer-related death.^[1]^ Esophageal squamous cell carcinoma (ESCC) accounts for over 90% of all EC cases in Asia. Due to the lack of specific symptoms associated with early-stage disease and robust biomarkers for early detection, treatment outcomes remain poor with a 5-year survival rate of only 15% to 25%.^[2]^ Patients with ESCC who are unsuitable for surgery or specific drug treatments are usually treated with chemo-radiotherapy (CRT).^[3,4]^ Also, many patients with early ESCC who are unwilling to receive surgery receive CRT or radiotherapy (RT) alone, as recommended by current evidence-based medicine guidelines. In clinical practice, tumor-node-metastasis (TNM) staging is often used to predict patient outcomes. However, there is a critical need for developing predictive biomarkers that can more accurately predict individual responses to therapy and inform the clinical management of patients with ESCC.

Evidence from research and clinical studies has highlighted the critical role of the immune system in tumor development and response to therapy.^[5]^ Total lymphocyte count (TLC) is considered an essential indicator of the immune response, and reduced lymphocyte counts are associated with poor prognosis in EC and other cancer types.^[6–8]^ Neutrophils are the primary source of angiogenesis-related and other growth factors in the circulatory system that play central roles in promoting tumor growth. Consequently, a higher neutrophil-lymphocyte ratio (NLR) indicates higher levels of neutrophils and lower lymphocytes in the patient indicating suppression of inherent immune response.^[9–11]^

Platelets can act to accelerate tumor growth and invasion by releasing platelet-derived angiogenic factors, which in turn increases the risk of death in cancer patients.^[12–14]^ Inflammatory biomarkers associated with prognosis in esophageal and other cancer types include a platelet-lymphocyte ratio (PLR) and lymphocyte-monocyte ratio (LMR).^[15–19]^ Lymphocyte, neutrophil and platelet levels can be easily measured by routine laboratory analyses which are economical and can be measured longitudinally at diagnosis and during treatment. However, temporal changes in hematological biomarkers are yet to be longitudinally evaluated and correlated with the radiosensitivity of ESCC patients receiving RT.

In this study, we aimed to determine changes in the levels of hematological biomarkers (NLR, PLR, LMR, and TLC) reflecting variations in immune function and individual radiosensitivity of ESCC patients.

## 2. Material and methods

### 2.1. Patients

Patients included in this study were diagnosed with ESCC and treated at the Affiliated Hospital of Jining Medical University from 2015 to 2019. Patients were diagnosed with local EC or were unable to undergo surgical treatment. The inclusion criteria for the study were: pathologically confirmed ESCC, receipt of ≥50 Gy RT with or without chemotherapy, Eastern Cooperative Oncology Group performance status of 0 to 2, available information from clinicopathological and laboratory data before treatment (RT alone or CRT), and complete imaging data. The exclusion criteria for the study included: distant metastasis, patients who had received palliative RT or re-irradiation, esophagography showing signs of esophageal perforation, and autoimmune, blood and infectious diseases or fever.

After screening, 353 patients were eligible for this study. All patients were evaluated by computed tomography, esophagogram, esophagogastroduodenoscopy, and other tests before initiation of treatment. The staging classification of EC was reviewed according to the 8th edition of the American Joint Committee on Cancer guidelines (AJCC8th edition). The Ethics Committee of the Affiliated Hospital of Jining Medical University approved this study and informed consent was obtained from the patients.

### 2.2. Treatment

All patients received RT alone or concurrent RT delivered using 3D-conformal radiotherapy (3D-CRT) or intensity-modulated radiotherapy. RT alone, radical simultaneous CRT or sequential CRT was performed according to clinical staging.

Target volumes and organs at risk were defined according to the Radiotherapy and Oncology Group guidelines. All plans were generated using the Eclipse Treatment Planning System (Version 13.5.35; Varian Medical Systems, Palo Alto, CA), and delivered with 6 MV photons beams. Prescribed doses were 50 to 66 Gy in 1.8 to 2.0 Gy fractions delivered once daily for 5 fractions per week. Plans were normalized to 95% of the plan tumor volume received 100% of the prescribed dose. The dose of all organs at risk was controlled within established normal tissue dose constraints.

Chemotherapy consisted of 2 platinum-based regimens; Cisplatin (25 mg/m^2^, days 1–4) with fluorouracil (700 mg/m^2^, days 1–4), and cisplatin with paclitaxel (135–175 mg/m^2^, day 1). Chemotherapy doses and regimens followed the guidelines of Chinese Society of Clinical Oncology, and the National Comprehensive Cancer Network for EC.

### 2.3. Response evaluation

Computed tomography and esophagograms were used to assess tumor response at 1 month after treatment. Tumor response was evaluated using the Response Evaluation Criteria in Solid Tumors guidelines (version 1.1). Patients who displayed complete response (CR) or partial response, were defined as RT sensitive. Patients who demonstrated stable disease or progressive disease (PD) were considered non-sensitive. Sensitivity can be observed in Figure S1, Supplemental Digital Content, http://links.lww.com/MD/I670.

### 2.4. Collection of clinical data

Clinicopathological and imaging data laboratory examinations were obtained from patient medical records. Clinicopathological data included sex, age, TNM stage, autoimmune history, treatment characteristics, Eastern Cooperative Oncology Group score, and tumor location. Complete blood counts were collected for laboratory examinations 1 week before RT. Neutrophil, total lymphocyte, platelet and monocyte counts were used to calculate NLR, PLR, and LMR values, respectively (NLR = neutrophil counts/TLC; PLR = platelet counts/TLC; LMR = TLC/ monocyte counts).

### 2.5. Statistical analysis

Descriptive statistics were used to summarize the clinicopathological and treatment characteristics of patients. Receiver operating characteristics (ROC) curves were used to determine the cutoff values for each hematological marker associated with radiosensitivity, and patients were divided into high and low groups. Statistical differences between measurement variables were analyzed using a Mann–Whitney *U* test. Statistical differences between enumeration variables were analyzed using the χ^2^ and Fisher exacts tests. Univariable logistic regression analysis was used to analyze the relationship between clinicopathological characteristics and radiosensitivity. Variables with *P* values <.10 in univariable analysis were included in the multivariable analysis.

Correlation analyses of NLR, PLR, LMR and TLC were performed using the Spearman’s correlation test. Statistically significant was determined at the level of *P* < .05. A nomogram was developed based on the multivariable logistic regression analysis results. All statistical analyses were performed using the Statistical Package for Social Sciences version 22.0 (SPSS, Chicago, IL) and R software (version 3.6.3; R Foundation for Statistical Computing, Vienna, Austria).

## 3. Results

### 3.1. Patient characterizes

The characteristics of patients recruited to the study are summarized in Table 1. Of the 353 total cases, 249 (70.5%) were male and 104 (29.5%) were female. The median age at treatment was 69 years [interquartile range, 63–75 years]. More than half of the patients in the study were treated with concurrent RT (56.4%). The number of patients with tumors located in the cervical, upper, middle and lower esophagus was 38 (13.2%), 83 (28.8%), 75 (10.4%), and 92 (31.9%), respectively. 108 (30.6%) patients had II diseases, 172 (48.7%) had stage III disease and 73 (20.7%) patients had stage IVA disease. 288 (81.6%) had cT stage 2 to 3 disease, 96 (27.2%) had cN stage 0 disease, and 176 (49.9%) had received ≥60 Gy of RT.

**Table 1:** Baseline characteristics of all 353 patients with ESCC.

Characteristics	All patients	Tumor responses		*P* value
		Sensitive	Non-sensitive	
No. of patients (%)	353 (100)	232 (65.7)	121 (34.3)	
Gender				
Male	249 (70.5)	158 (68.1)	91 (75.2)	.165
Female	104 (29.5)	74 (31.9)	30 (24.8)	
Age				
<70	180 (51.0)	121 (52.2)	59 (48.8)	.545
≥70	173 (49.0)	111 (47.8)	62 (51.2)	
cT stage				
2–3	288 (81.6)	186 (80.2)	102 (84.3)	.343
4	65 (18.4)	46 (19.8)	19 (15.7)	
cN stage				
0	96 (27.2)	61 (26.3)	35 (28.9)	.598
1–3	257 (72.8)	171 (73.7)	86 (71.1)	
TNM stage				
II–III	280 (79.3)	181 (78.0)	99 (81.8)	.403
IVA	73 (20.7)	51 (22.0)	22 (18.2)	
Location				
Cervical	49 (13.9)	37 (15.9)	12 (9.9)	.322
Upper	96 (27.2)	58 (25.0)	38 (31.4)	
Middle	98 (27.8)	63 (27.2)	35 (28.9)	
Lower	110 (31.2)	74 (31.9)	36 (29.8)	
Treatment				
RT alone	154 (43.6)	100 (43.1)	54 (44.6)	.784
RT + concurrent	199 (56.4)	132 (56.9)	67 (55.4)	
RT dose				
50–60	177 (50.1)	121 (52.2)	56 (46.3)	.295
≥60	176 (49.9)	111 (47.8)	65 (53.7)	
NLR, median (IQR)	2.35 (1.69–3.27)	1.99 (1.46–2.69)	3.16 (2.35–3.98)	<.001
PLR, median (IQR)	139.7 (109.7–186.7)	128.7 (101.3–172.7)	168.8 (130.0–210.1)	<.001
LMR, median (IQR)	3.44 (2.63–4.55)	3.67 (2.93–4.86)	2.98 (2.16–3.98)	<.001
TLC, ×10³/μL, median (IQR)	1.66 (1.28–2.17)	1.70 (1.33–2.25)	1.60 (1.16–1.95)	.009

cN = clinical lymph node, cT = clinical tumor, ESCC = esophageal squamous cell carcinoma, IQR = interquartile range, LMR = lymphocyte-monocyte ratio, NLR = neutrophil-lymphocyte ratio, PLR = platelet-lymphocyte ratio, RT = radiotherapy, TLC = total lymphocyte count, TNM = tumor-node-metastasis.

ROC curve analysis showed the cutoff value for pretreatment NLR was 2.59, with a sensitivity of 69.4%, and a specificity of 72.4%. The data had an area under the curve of 0.752. The optimal cutoff values for the other pretreatment hematological markers were 142.9 for PLR, 2.78 for LMR, 1.96 × 10^3^/μL for TLC. Patients were divided into high and low subgroups based on these cutoff values, and radiosensitivity was analyzed according to these stratifications.

### 3.2. Pretreatment hematological markers and clinical characterizes

Pretreatment hematological biomarkers (NLR, PLR, LMR, and TLC) could predict tumor responses as shown in Table 2. Following treatment, 232 (65.7%) patients were radiosensitive consisting of 166 (81.8%) of 203 patients in the NLR low-count group (<2.59) and 66 (44%) of 150 patients in the NLR high-count (≥2.59) group, as shown in Table 1. Patients with high and low NLRs had significantly different baseline characteristics in terms of cT-stage, as shown in Table 2. Excluding these characteristics, no other patient-specific parameters (gender, age, cN stage, TNM stage, location, treatment, and RT dose) were associated with significant differences between the 2 groups.

**Table 2:** Comparison of pretreatment hematological markers and clinical characteristics in all 353 ESCC patients.

Characteristics	NLR			PLR			LMR			TLC*		
	<2.59	≥2.59	*P*	<142.9	≥142.9	*P*	<2.78	≥2.78	*P*	<1.96	≥1.96	*P*
Gender												
Male	147	102	.369	127	122	.514	72	177	.867	170	79	.118
Female	56	48		57	47		31	73		62	42	
Age (yr)												
<70	100	80	.449	98	82	.373	55	125	.562	109	71	.037
≥70	103	70		86	87		48	125		123	50	
cT stage												
2–3	158	130	.034	147	141	.391	95	193	.001	193	95	.282
4	45	20		37	28		8	57		39	26	
cN stage												
0	56	40	.678	55	41	.235	36	60	.036	65	31	.631
1–3	102	81		129	128		67	190		167	90	
TNM stage												
II–III	154	126	.062	143	137	.438	92	188	.003	186	94	.584
IV	49	24		41	32		11	62		46	27	
Location												
Cervical	29	20	.984	23	26	.824	11	38	.693	31	18	.681
Upper	55	41		52	44		28	68		59	37	
Middle	55	43		53	45		29	69		67	31	
Lower	64	46		56	54		35	75		75	35	
Treatment												
RT alone	91	63	.596	79	75	.785	47	107	.626	109	45	.078
RT + concurrent	112	87		105	94		56	143		123	76	
RT dose, Gy												
50–60	104	73	.634	101	76	.063	49	128	.536	114	63	.601
≥60	99	77		83	93		54	122		118	58	
Tumor responses												
Sensitive	166	66	<.001	144	88	<.001	49	183	<.001	140	92	.003
Non-sensitive	37	84		40	81		54	67		92	29	

cN = clinical lymph node, cT = clinical tumor, ESCC = esophageal squamous cell carcinoma, LMR = lymphocyte-monocyte ratio, NLR = neutrophil-lymphocyte ratio, PLR = platelet-lymphocyte ratio, Pre- = pretreatment, RT = radiotherapy, TLC = total lymphocyte count, TNM = tumor-node-metastasis.

*× 10³/μL.

The PLR response was similar to that of NLR with 144 (78.3%) patients in the low radiosensitivity group, compared to 88 (52.1%) in the high radiosensitivity group. Also, 183 (73.2%) patients were radiosensitive in the high LMR group, while 54 (52.4%) patients in the low PLR group were not radiosensitive (*P* value <.001). Patients with low and high radiosensitivity had significantly different baseline characteristics in cT, cN, and TNM stages as shown in Table 2. No statistically significant correlation was observed with the other characteristics between the different LMR groups. Also, differences in age between the TLC groups were observed which may be due to impaired lymphatic function in elderly patients.

### 3.3. Uni- and multivariate analyses of tumor response

The results of the univariate logistic regression analysis are presented in Table 3. The factors potentially associated with tumor response include high NLR (odds ratio [OR] 5.710, 0.95 confidence interval [CI] 3.531–9.233, *P* < .001) and high PLR (OR 3.314, 0.95 CI 2.087–5.262, *P* < .001) as being associated with worse RT sensitivity. High LMR (OR 0.332, 0.95 CI 0.206–0.535, *P* < .001), high TLC (OR 0.480, 0.95 CI 1.633–5.691, *P* < .001) were associated with lower radiosensitivity. Spearman correlation analysis (shown in Figure S2, Supplemental Digital Content, http://links.lww.com/MD/I671) showed that pretreatment NLR was positively correlated with PLR (*R* = 0.638, *P* < .001), and negatively correlated with LMR (*r* = −0.698, *P* < .001). Due to the overestimation of standard errors by collinearity, these variables cannot be simultaneously examined and require testing in the multivariate analysis. Multivariate analysis showed that high NLR (OR 5.693, 0.95 CI 3.500–9.260, *P* < .001) and high PLR (OR 3.305, 0.95 CI 2.070–5.279, *P* < .001) were independently associated with of high radiosensitivity Table 4. Two other elements the high LMR (OR 0.326, 0.95 CI 0.201–0.530, *P* < .001) and high TLC were also found to be independently associated with higher radiosensitivity in multivariate analysis.

**Table 3:** Univariate analysis of factors associations between clinical characteristics and tumor response in all 353 patients with ESCC.

Variable	OR	95% CI	*P* value
Gender (male vs female)	0.704	0.428–1.156	.166
Age (<70 vs ≥70)	1.146	0.738–1.778	.545
cT stage (2–3 vs 4)	0.753	0.419–1.354	.344
cN stage (0 vs 1–3)	0.877	0.537–1.430	.598
TNM stage (II–III vs IVA)	0.789	0.452–1.376	.403
Location			
Cervical	Ref.		.329
Upper	2.020	0.936–4.358	.073
Middle	1.713	0.792–3.704	.171
Lower	1.500	0.699–3.218	.298
Treatment (alone vs concurrent)	0.940	0.604–1.463	.784
RT dose (50–60 vs ≥60)	1.265	0.814–1.966	.295
NLR (<2.59 vs ≥2.59)	5.710	3.531–9.233	<.001
PLR (<142.9 vs ≥142.9)	3.314	2.087–5.262	<.001
LMR (<2.78 vs ≥2.78)	0.332	0.206–0.535	<.001
TLC, ×10³/μL (<1.96 vs ≥1.96)	0.480	0.293–0.789	.004

CI = confidence interval, cN = clinical lymph node, cT = clinical tumor, ESCC = esophageal squamous cell carcinoma, LMR = lymphocyte-monocyte ratio, NLR = neutrophil-lymphocyte ratio, OR = odds ratio, PLR = platelet-lymphocyte ratio, RT = radiotherapy, TLC = total lymphocyte count, TNM = tumor-node-metastasis.

**Table 4:** Multivariate analysis of factors associations between clinical characteristics and tumor response in all 353 patients with ESCC.

Variable	OR	95% CI	*P* value
Model 1			
TLC, ×10³/μL (<1.96 vs ≥1.96)	0.483	0.284–0.823	.007
NLR (<2.59 vs ≥2.59)	5.693	3.500–9.260	<.001
Model 2			
TLC, ×10³/μL (<1.96 vs ≥1.96)	0.482	0.289–0.804	.005
PLR (<142.9 vs ≥142.9)	3.305	2.070–5.279	<.001
Model 3			
TLC, ×10³/μL (<1.96 vs ≥1.96)	0.467	0.281–0.777	.003
LMR (<2.78 vs ≥2.78)	0.326	0.201–0.530	<.001

CI = confidence interval, ESCC = esophageal squamous cell carcinoma, LMR = lymphocyte-monocyte ratio, NLR = neutrophil-lymphocyte ratio, OR = odds ratio, PLR = platelet-lymphocyte ratio, TLC = total lymphocyte count.

### 3.4. Development of a predictive nomogram for radiosensitivity

**Figure 1.**
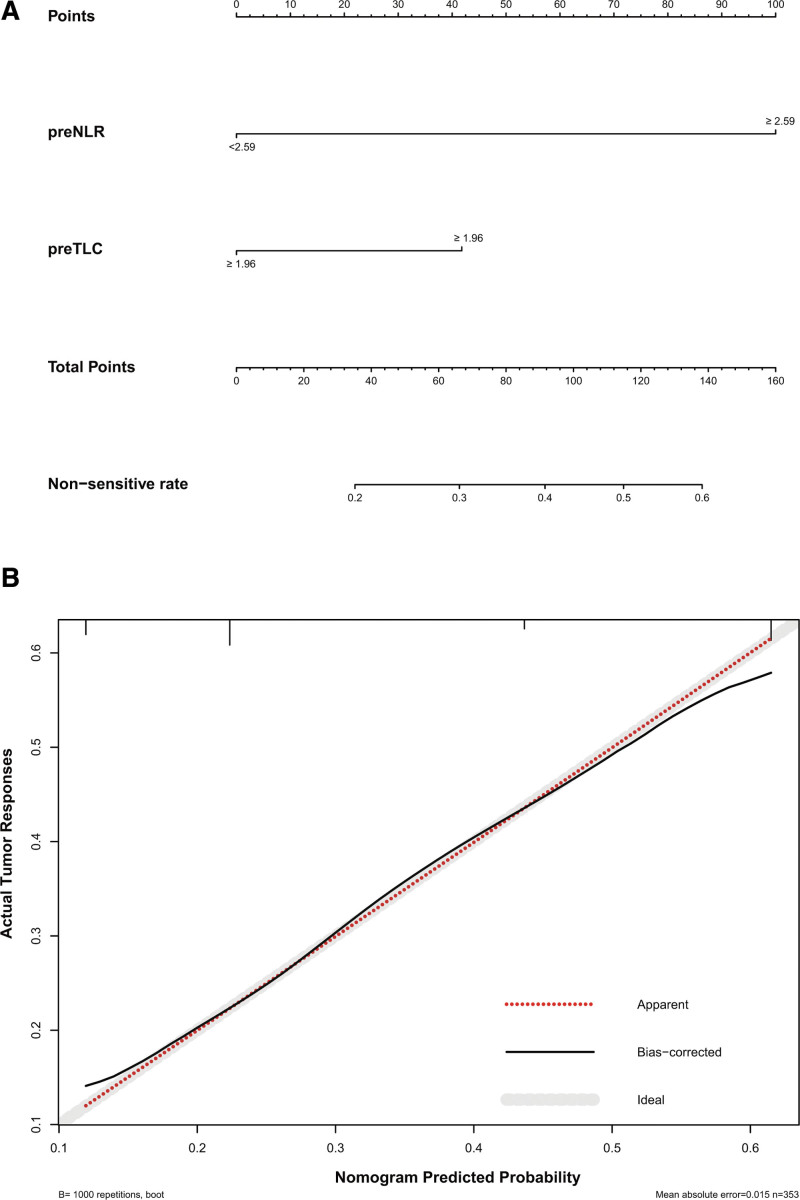
Nomogram predicting the tumor responses (A) and calibration curves (B).

A nomogram capable of predicting patient radiosensitivity was established based on the results of the multivariate analysis (Fig. 1A). In particular, Model 1 had a concordance index of 0.737 (95% CI 0.628–0.792), which was better than the other models (Model 2: 0.681, 95% CI 0.622–0.739; Model 3: 0.656, 95% CI 0.596–0.717). The calibration curves for the tumor-sensitive nomograms demonstrated good agreement between predictions and actual experimental observations (Fig. 1B).

## 4. Discussion

In this study, we retrospectively analyzed a series of newly diagnosed patients with ESCC who underwent routine blood tests before treatment. Pretreatment NLR, PLR, LMR, and TLC can be used as valid indicators to predict tumors’ response to radiation therapy in patients with ESCC. In addition, we developed and validated a visualized nomogram that can be used clinically by physicians and patients, and was validated to provide early prediction of sensitivity in ESCC patients treated with RT.

Overall poor prognosis in ESCC patients may be due to the strong biological ability of ESCC tumor cells to proliferate and invade surrounding tissues. The esophagus has an abundance of lymphatic vessels in which cells can disseminate. Also, there is a greater number of longitudinally arranged vessels than those in the transverse arrangement, resulting in earlier lymph node metastasis.^[20,21]^

For patients with inoperable EC, RT is the primary treatment option. However, around 27% to 50% of patients experience local recurrence, and metastasis or both after treatment.^[22,23]^ OS was significantly worse in patients who were less sensitive to RT compared to more radiosensitive patients. Patients who were re-radiated had side effects related to esophageal fistula, pulmonary fibrosis, perforation and cardiotoxicity. Therefore, more reliable biomarkers are needed to predict response to RT in patients with EC and minimize toxicities resulting from treatment.

In 1863, Virchow detected leukocytes in tumor samples and suggested an interrelationship between tumor development and the immune response. Subsequently, this relationship has attracted significant research interest demonstrating the complex interactions between the immune system and tumor within the tumor microenvironment.

Inflammatory cells secrete cytokines (e.g., IL-6, TNF-α, and TGF-β) that affect tumor cell growth, development, and metastasis.^[24]^ Inflammatory biomarkers including NLR and PLR can be easily determined and clinical studies have shown NLR is associated with prognosis in multiple cancers, including EC.^[25–29]^

In a study of neoadjuvant chemotherapy for breast cancer, better pathological CR was found in patients with a pretreatment NLR < 2.06. Also, NLR is an independent predictor of relapse-free survival.^[25]^ High NLR in patients with pancreatic head cancer (>2.7) and EC (>2.64) is associated with poor prognosis.^[26,27]^ Furthermore, the relationship between NLR and RT efficacy was retrospectively analyzed in 56 patients with cervical squamous carcinoma treated with either RT alone, or concurrent RT. The CR rate in the low NLR group versus the high NLR group (81.0% vs 22.9%, *P* < .001) was different. In addition, OS and PFS rate in the low NLR group was higher in the high NLR group (59.7% vs 35.9%, *P* = .04; 72.4% vs 37.7%, *P* < .01).^[28]^ Finally, in a phase 3 clinical trial with long-term follow-up of metastatic pancreatic cancer treatment, statistically significant improvements in OS were observed in patients with a baseline NLR ≤ 5 (9.1 months vs 5.0 months, OR = 1.839, *P* < .001).^[29]^

In conclusion, the above experimental and clinical results suggest that pretreatment NLR impacts patient efficacy and survival after treatment. We analyzed and confirmed pretreatment NLR, PLR, LMR, and TLC as predictors of radiosensitivity by univariate logistic regression. Due to the strong co-collinearity of NLR, PLR, and LMR, each was tested separately in multivariate logistic regression analysis. Results showed NLR, PLR, LMR, and TLC were independent risk factors for RT sensitivity. Finally, we established a nomogram that could predict patient radiosensitivity based on model 1, which performed well in predicting response and was supported by the concordance index, and the calibration curve.

Our results showed that pretreatment blood biomarkers in ESCC patients could be used to predict radiosensitivity, in agreement with previous data from Zhang et al.^[30]^ In this study, an analysis of 266 patients with ESCC showed response rates of 89.3% in the low NLR group (<3.06), and 68% in the high NLR group. Tang et al^[31]^ reported that lower lymphocytes during RT were associated with poorer patient prognosis, and worse OS. However, the recovery of lymphocytes is not associated with improved prognosis.^[32]^ Neutrophils are an integral part of the innate immune system which kills foreign pathogens to protect the host. Neutrophils can also trap pathogens through neutrophil extracellular traps. Recently, neutrophil extracellular traps have been found to mediate enhanced invasiveness and metastasis in cancer cells.^[33,34]^ These observations highlight that ESCC patients with higher systemic inflammation indices are less radiosensitive. This may be due to infiltrating immune cells in the tumor tissue secreting IL-6, IL-8, IL-1β, TNF-α, TGF-β and other cytokines, during RT which in turn promote the growth and movement of tumor cells.^[35–37]^

In a retrospective analysis of ESCC, Chen et al^[38]^ found that response to treatment in patients with higher IL-6 levels was significantly associated with decreased OS. Furthermore, it was shown that inhibition of IL-6 gene expression improved patient response to RT and the biological behavior of the tumor. Similar findings have been observed in prostate cancer patients.^[39]^ IL-8 belongs to the CXC chemokine superfamily that promotes inflammation by activating neutrophils and other immune cells, and can promote tumor angiogenesis.^[40,41]^ In addition, RT induces the expression and activation of IL-1β and TGF-β. In a breast cancer study, Paquette et al^[42]^ found that patient IL-1β levels were elevated after completion of RT which could induce the invasive capacity of cancer cells. Also, IL-1β may affect the above mechanisms through the expression of matrix metalloproteinase-9 and COX-2.

TGF-β may induce mesenchymal transformation to enhance cancer cells’ invasion and metastasis; however, the exact mechanism remains to be entirely determined.^[43,44]^ After patients receive RT, both anti- and pro-inflammatory factors dynamically change with cytokines interacting with the tumor microenvironment in a complex cytokine network.

Our study also had several limitations. Firstly, this was a retrospective study in a single center, and heterogeneity of laboratory tests for hematological parameters was observed. Secondly, we used ROCs to determine the optimal cutoff value, yet there currently needs to be a precise way to determine these values for inflammatory indicators. Finally, not all blood inflammatory markers were assessed in this analysis, and other factors such as CRP and interleukin should be evaluated. Our data require further randomized studies to validate the presented experimental results.

## 5. Conclusion

Hematological markers (NLR, PLR, LMR, and TLC) before RT in patients with ESCC are independent factors that affect radiosensitivity. Based on the results of multivariate analysis, we created a nomogram that was internally validated and showed good agreement with experimental findings. This visualization tool can assist with patient counseling and treatment strategies adjustment and may also be used to guide individualized treatment decisions in clinical practice.

## Author contributions

**Conceptualization:** Yanhui Zhang.

**Data curation:** Peng Wei, Shuang Ge, Jie Zheng.

**Funding acquisition:** Shucheng Ye.

**Investigation:** Peng Wei, Shuang Ge.

**Methodology:** Shuang Ge, Shucheng Ye.

**Supervision:** Shucheng Ye, Yanhui Zhang.

**Writing – original draft:** Lijun Sun.

**Writing – review & editing:** Lijun Sun, Yanhui Zhang.

## Supplementary Material
