# Supplementary material for: A nomogram based on hematological markers to predict radiosensitivity in patients with esophageal squamous cell carcinoma
Source: Medicine (Baltimore). 2023 Mar 17;102(11):e33282. doi: 10.1097/MD.0000000000033282 (PMC10019115; doi:10.1097/MD.0000000000033282)
Supplement: Supplementary file 1 [file medi-102-e33282-s001.pdf]

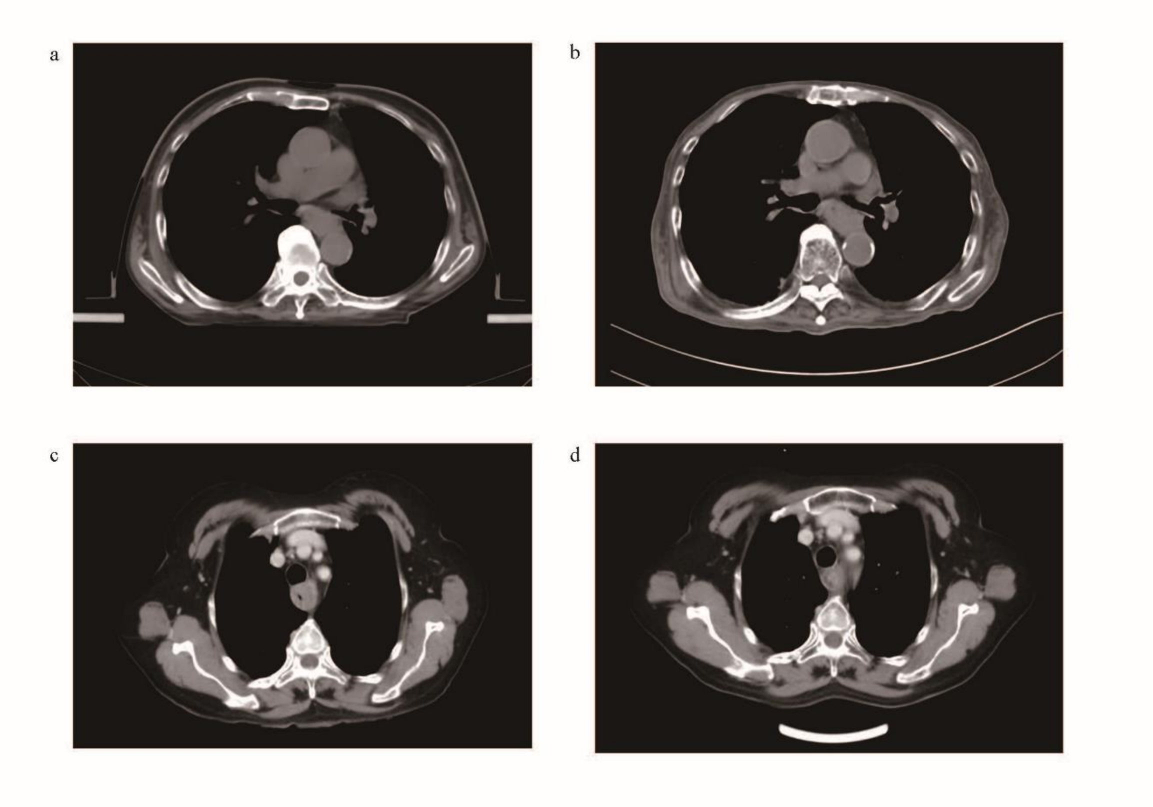

Supplementary Figure 1. Treatment of insensitive and sensitive patients. (a) Imaging tests were conducted on Patient A prior to the initiation of treatment. (b) The imaging examination of patient A one-month post-treatment. (c) Imaging tests were conducted on Patient B prior to the initiation of treatment. (d) The imaging examination of patient B one-month post-treatment.
