# Supplementary material for: A nomogram based on hematological markers to predict radiosensitivity in patients with esophageal squamous cell carcinoma
Source: Medicine (Baltimore). 2023 Mar 17;102(11):e33282. doi: 10.1097/MD.0000000000033282 (PMC10019115; doi:10.1097/MD.0000000000033282)
Supplement: Supplementary file 2 [file medi-102-e33282-s002.pdf]

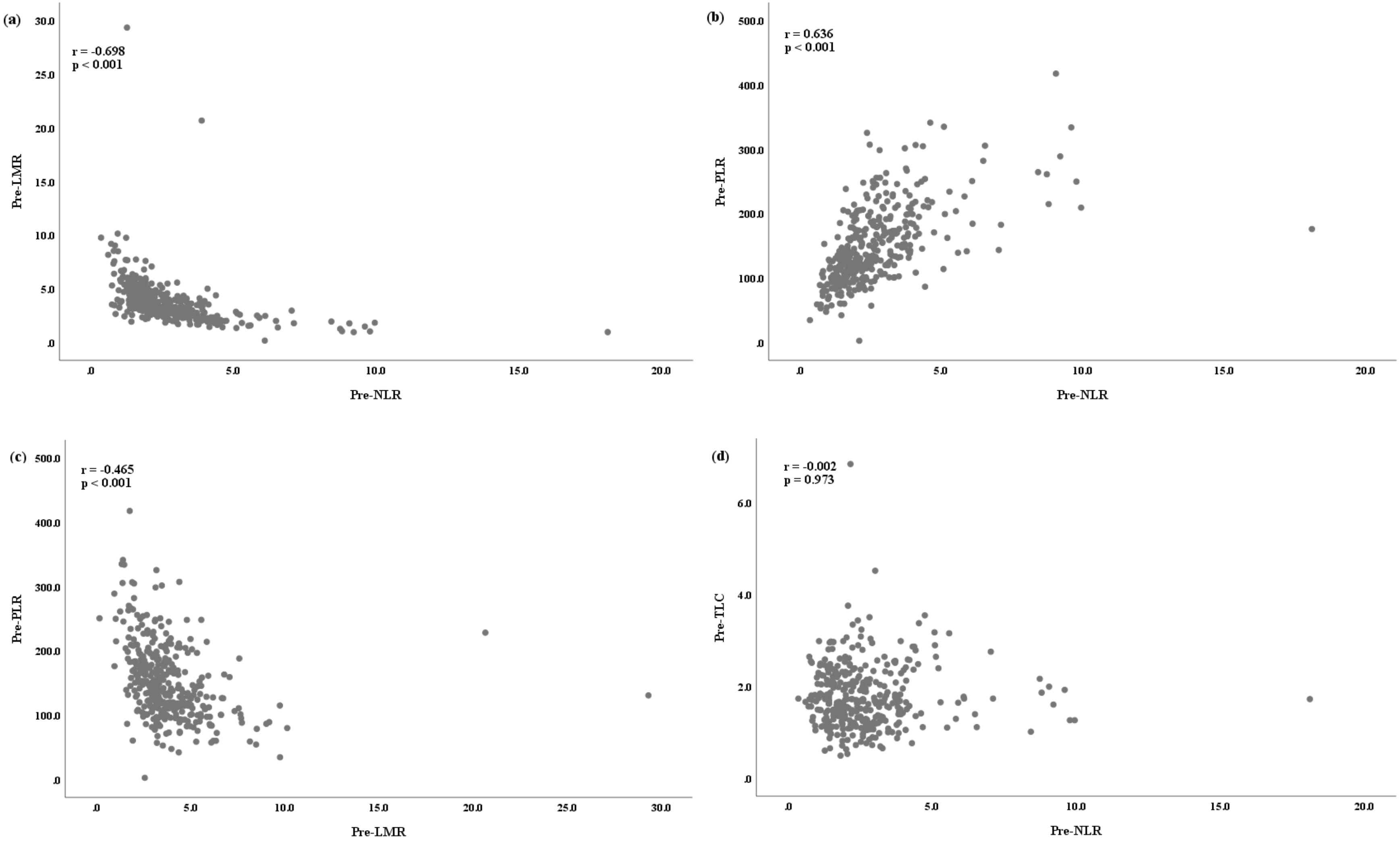
(a)
r = -0.698
p < 0.001
Pre-LMR
Pre-NLR
(b)
r = 0.636
p < 0.001
Pre-PLR
Pre-NLR
(c)
r = -0.465
p < 0.001
Pre-PLR
Pre-LMR
(d)
r = -0.002
p = 0.973
Pre-TLC
Pre-NLR

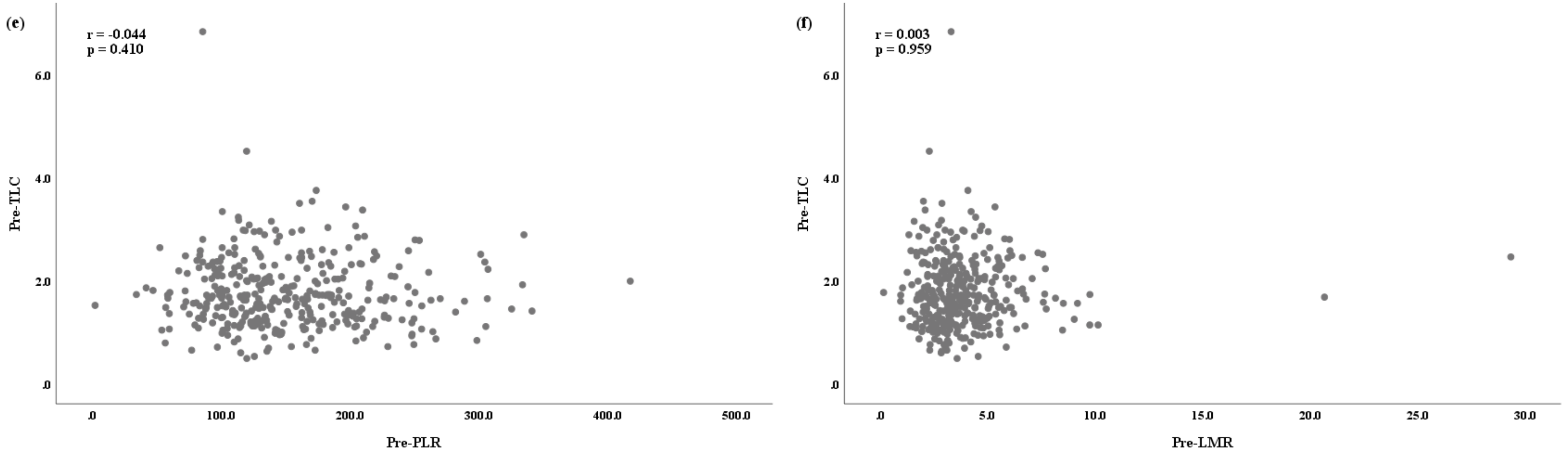

Supplementary Figure 2：Spearman’s correlation analyses of NLR, PLR, LMR and TLC.

Abbreviations: Pre-, Pretreatment; LMR, lymphocyte-monocyte ratio; NLR, neutrophil-lymphocyte ratio; PLR, platelet-lymphocyte ratio; TLC, total lymphocyte count.
